# While We Wait for a Vaccine Against SARS-CoV-2, Why Not Think About Available Drugs?

**DOI:** 10.3389/fphys.2020.00820

**Published:** 2020-07-03

**Authors:** Francisco J. Barrantes

**Affiliations:** Biomedical Research Institute (BIOMED), Argentina Pontifical Catholic University of Argentina (UCA) and National Scientific and Technical Research Council (CONICET), Buenos Aires, Argentina

**Keywords:** coronavirus, COVID-19, SARS-CoV-2, design drugs, ACE2, prophylaxis

## Abstract

At the time of reception of this article (April 2, 2020), efforts to develop a specific vaccine against SARS-Cov-2, the causative agent of the coronavirus disease 2019 (COVID-19), had just begun trial phase 1, but full validation of this and other current developments is likely to take many more months to reach completion. The ongoing pandemic constitutes a major health burden of world proportions that is also having a devastating impact on whole economies worldwide, the knock-on effects of which could be catastrophic especially in poorer countries. Alternative measures to ameliorate the impact and hamper or minimally slow down disease progression are urgently called for. This review discusses past and currently evolving data on the etiological agent of the current pandemic, SARS-CoV-2, and its host cell receptors with a view to disclosing alternative drugs for palliative or therapeutic approaches. Firstly, SARS-CoV-2 exhibits marked tropism for cells that harbor the membrane-bound metalloprotease angiotensin-converting enzyme 2 (ACE2) at their plasmalemma, predominantly in cells lining the oral cavity, upper respiratory tract, and bronchoalveolar cells, making these epithelial mucosae the most likely viral receptor cell targets and entry routes. Secondly, the crystal structures of several coronavirus spike proteins in complex with their cell host target receptors, and of SARS-Cov-2 in complex with an inhibitor, are now available at atomic resolution through X-ray diffraction and cryo-electron microscopy studies. Thirdly, viral entry of other viruses has been successfully blocked by inhibiting viral endogenous proteases or clathrin/dynamin-dependent endocytosis, the same internalization pathway followed by ACE2 and some viruses. Fourthly, the target cell-surface receptor molecules and SARS-CoV-2 possess other putative sites for drugs potentially modulating receptor activity or virus processing. A multi-pronged pharmacological approach attacking more than one flank of the viral-receptor interactions is worth considering as a front-line strategy.

## Introduction

Human coronaviruses (HCoVs) were discovered in 1965 in patients with the common flu and coined B814, with the prefix “corona” subsequently added in reference to their relatively large spikes (or spines) resembling a crown (Li, 2013). HCoVs belong to the family of Coronaviridae enveloped viruses that harbor between 26 and 32 kilobases of single-stranded positive-sense RNA, the largest so far observed for an RNA virus (Li, 2013; Su et al., 2016), enveloped in a sphere of 80–120 nm in diameter. CoVs infect a wide spectrum of avian and mammalian species. Seven human CoVs are currently known. Members of the first group (HCoV-OC43, HCoV-293, HCoV-NL63, and HKU1-CoV) circulate in humans and generally cause mild, self-limiting respiratory diseases. HCoVs in the second group are more pathogenic and share the tropism for epithelial cells containing membrane bound proteases; they are the etiological agents responsible for the severe acute respiratory syndrome (SARS-CoV), Middle East respiratory syndrome (MERS-CoV), and the ongoing outbreak of CoV disease (COVID-19) purportedly originating in the city of Wuhan, Hubei province in China. The etiological agent, SARS-CoV-2, is a beta-CoV that has been isolated from human bronchoalveolar epithelium of infected patients (Zhou P. et al., 2020). Full-genome sequencing of SARS-CoV-2 showed that it has 79.5% sequence identity with SARS-CoV and is 96% identical to the Chinese bat CoV, BatCoV RaTG13 (Zhou P. et al., 2020).

CoVs are related zoonotically, sharing common phylogeny and structural properties but showing differences in tropism, host range, cell-surface receptors, mechanisms for entry into cells, etiopathology, clinical presentation and epidemiological characteristics. In humans, CoV infections predominantly affect the respiratory and gastrointestinal tracts. The potential emergence of a SARS-like cluster of a circulating bat CoV, SHCO14-CoV, with experimentally demonstrated human cell infective capacity and high pathogenicity, was already reported in 2015 (Menachery et al., 2015). The authors warned of the potential risk of SARS-CoV re-emergence. Analysis of evolutionary, genetic, and pathogenic aspects of CoVs reiterated the warning (Su et al., 2016): “considering the high frequency of recombination of these viruses, with unpredictable changes in virulence, and with multiple viral species hosted by different animals that are likely to interact with each other, it is not a matter of if, but of when, a new CoV will emerge and cause a new outbreak of human disease.” The current, perhaps inevitable outbreak of COVID-19 attests to the correctness of these predictions.

## Infection: Viral Recognition and Entry Into Target Cells

Coronavirus possesses four main structural proteins [nucleocapsid (N), spike (S), envelope (E), and membrane (M)] and various non-structural proteins (nsp). The surface spike S glycoprotein plays the key role in addressing and infecting the cell containing target membrane-bound receptors. Virion entry is a multi-step process involving attachment to the cell surface, receptor engagement, protease processing and membrane fusion. The SARS-CoV enters human epithelial cells *in vitro* through the apical surface, and viruses replicated in these cells are also released via the apical plasmalemma. This correlates with the cellular distribution of its main molecular target at the plasmalemma, the angiotensin-converting enzyme 2 (ACE2), which in well-differentiated epithelial cells is more abundantly expressed at the apical surface than in the basolateral membranes and is used by SARS-CoV to readily infect such differentiated cells (Jia et al., 2005). Based on this evidence, and the phylogenetic kindredness among CoVs, it was highly likely that SARS-CoV-2 would also follow the same entry route, as recently demonstrated (Zhou P. et al., 2020).

It has long been known that the viral spike S glycoprotein is critical for host range and tropism (Supekar et al., 2004; Li et al., 2005). The S protein is a trimer, consisting of three S1–S2 subunit heterodimers. During viral infection, each trimer is cleaved into its S1 and S2 subunits. The S1 N-terminal domain contains the receptor binding domain (RBD), which can bind a variety of targets including polypeptide segments of proteolytic enzymes like ACE2 or carbohydrate moieties like neuraminic acid or heparan sulfate. In mouse hepatitis virus (MHV) beta-CoV the S1 N-term domain is recognized by cell adhesion carcinoembryonic antigen-related cell adhesion molecule 1 (CEACAM1) (Kubo et al., 1994). Another zinc metalloprotease enzyme, aminopeptidase N (APN, CD13), acts as a viral-recognition protein for human H229E-CoV, transmissible gastroenteritis virus, porcine epidemic diarrhea, and feline infectious peritonitis virus. The enzyme dipeptidyl-peptidase 4 (DPP4), also known as a cluster of differentiation 26 (CD26), is found at the apical surface of unciliated bronchial epithelial cells and performs this function for MERS-CoV, a member of the beta-CoV genus which is not recognized by the ACE2 receptor, and which has a remarkable evolutionary ability to adapt to species variations in its DPP4 cell-surface target by modifying the S protein surface charge (Letko et al., 2018). In addition to using DPP4, MERS-CoV can infect human pulmonary epithelial cells through highly specific but low affinity interactions with sialic acid residues present in host cell-surface glycoproteins (Li W. et al., 2017) using a different region of its spike protein S (Park et al., 2019).

Regarding the putative participation of DPP4 in SARS-CoV-2 infection, the notion that ACE2 shows relatively low expression levels in alveolar cells led Zhang and coworkers (Qi et al., 2020) to explore the expression of other proteins that might act as co-receptors of SARS-CoV-2. Using single-cell gene expression matrices, DPP4 was found to have a similar expression pattern in multiple cells and tissues together with ACE2 and the peptidases ANPEP and ENPEP (Qi et al., 2020); however, no evidence was found of DPP4 acting as a co-receptor of SARS-CoV-2.

Carbohydrate moieties are likely to receive increasing attention because of their role in spike protein-cell receptor interactions. In a recent mass spectrometry study of a recombinant SARS-CoV-2 (Watanabe et al., 2020) the glycan moiety of the S glycoprotein was dissected in great detail. Each trimer of the S glycoprotein contains 66 N-linked glycosylation sites, which may participate in viral protein folding and stability, but also intervene in viral tropism. Furthermore, the glycosylation sites are under selective pressure, as they shield viral epitopes from being recognized and neutralized by host antibodies, thus facilitating immune evasion by a camouflage mechanism as observed with the HIV envelope glycoprotein (Sok et al., 2016).

To identify the putative receptor of SARS-CoV-2, sequence and phylogenetic analyses of other CoVs were carried out together with a molecular modeling exercise on the S protein of the SARS-CoV-2 virus. The results of these *in silico* studies led Xu et al. to formulate the hypothesis that ACE2 was the SARS-CoV-2 cell host receptor (Xu et al., 2020b). This contention was followed soon after by the experimental demonstration that ACE2 was indeed the cellular target of SARS-CoV-2 (Zhou P. et al., 2020). Remarkably, the S protein of HNL63-CoV, an alpha-CoV, is recognized by ACE2 (Li et al., 2005). It is apparent that CoVs exhibit predominant binding tropisms toward membrane-bound proteases, but such tropisms are by no means absolute.

Spike S protein-mediated binding of CoVs to the surface of the host cells and fusion to the cell-surface membrane appears to be a conserved, shared mechanism. It is hypothesized, however, that the two mechanisms may have evolved separately (Li, 2016): ancestral CoVs would have initially harbored a primordial spike with S2 domains only, functionally inefficient because the virus had to randomly diffuse to reach target cells. Membrane fusion eventually occurred in a receptor-independent manner. The spike would have subsequently evolved to acquire a galectin-like S1 N-term domain through gene capture, thus enhancing CoV efficiency in infecting cells. A third evolutionary stage is purported to be the appearance of an S1 C-term domain through gene duplication of the S1 N-term domain (Li, 2016). A comprehensive review on the binding-proteolytic activation-fusion mechanism of CoVs entry (Millet and Whittaker, 2015) emphasizes that the proteolytic activation step is the critical one for the fusion of S1 to occur, since it allows for controlled release of the fusion peptide into the target plasmalemma.

It should be noted that although ACE2 appears to be the main cell-host receptor employed by SARS-CoV-2 to invade cells, other putative receptor molecules have recently been proposed, such as the surface glycoprotein CD147, also known as EMMPRIN or Basigin, a member of the immunoglobulin superfamily, with peptide-9 (an antagonist of C147) inhibiting SARS-CoV-2 binding to HEK293 cells (Wang K. et al., 2020).

TMPRSS2 is a transmembrane serine protease expressed in several tissue. The gene coding for this enzyme, *TMPRSS2*, is the most frequently altered gene in primary prostate cancer (Stopsack et al., 2020). This bears relationship to COVID-19, because TMPRSS2 expression is regulated by sex hormones, and COVID-19 shows predominance in males (Grasselli et al., 2020; Guan et al., 2020; Richardson et al., 2020). TMPRSS2 activation is exploited by a variety of corona and filo-viruses and Ebola virus (Zhou et al., 2015), and some influenza and parainfluenza viruses (Laporte and Naesens, 2017) to activate the viral protein machinery involved in the fusion with the host cell plasmalemma. TMPRSS2 proteolytically activates MERS-CoV (Shirato et al., 2013) and SARS-CoV (Matsuyama et al., 2010) *in vivo* and *in vitro*. Most recently an engineered cell line, VeroE6/TMPRSS2 (VeroE6 cell line expressing TMPRSS2) was shown to be highly susceptible to SARS-CoV-2 infection, suggesting the important role of TMPRSS2 in SARS-CoV-2 infection and indicating its potential utility for isolating and propagating this virus (Matsuyama et al., 2020). The common mechanism of action of TMPRSS2 on CoVs is the activation or priming of the spike S proteins at the viral S1/S2 cleavage site (Shen et al., 2017; Hoffmann et al., 2020a). Similarly, TMPRSS2 is involved in the cleavage of the hemagglutinin surface protein in the case of some influenza viruses (Laporte and Naesens, 2017).

## ACE and ACE2 and Their Ying-Yang Roles in the Renin-Angiotensin- Aldosterone System (RAAS), Hypertension and Diabetes in COVID-19

Endothelial ACE is a key metallopeptidase enzyme in the renin-angiotensin-aldosterone system (RAAS), playing a crucial role in the regulation of blood pressure and homeostasis of body fluids. Human endothelial ACE catalyzes the removal of the carboxy-terminal dipeptide from the decapeptide angiotensin I to produce angiotensin II, the active vasoconstrictor form of the hormone. Angiotensin II and aldosterone are the two key biologically active hormones of the RAAS homeostatic system (Li X. C. et al., 2017; Mirabito Colafella et al., 2019). ACE2 is also a metalloprotease, initially described in a study searching for novel genes related to heart failure, and found in membrane-associated and secreted forms predominantly in endothelial cells of human heart, kidney, and testis (Donoghue et al., 2000). Endothelial ACE and ACE2 share 42% identical amino acid residues in their catalytic domain, suggesting a common ancestor (Donoghue et al., 2000).

Avian influenza H5N1 virus, phylogenetically unrelated to the CoVs, appears to affect the RAAS system: infected patients exhibit higher levels of angiotensin II in serum, a sign which correlates with the severity and lethality of the disease. Furthermore, parallel studies conducted in animal models showed that disease severity correlates with the downregulation of ACE2 in lung, which can be reversed, increasing animal survival, by administration of recombinant human ACE2 (Zou et al., 2014).

Early epidemiological observations indicated higher morbidity and mortality among elderly Chinese COVID-19 patients with hypertension and diabetes (Wu et al., 2020; Zhou F. et al., 2020). In a cohort of 416 Chinese patients with COVID-19 about 60% had hypertension and ~20% had cardiac injury (Shi et al., 2020). Patients with severe COVID-19 and diabetes or hypertension as comorbidities were advised not be treated with ACE inhibitors or angiotensin IIAT1 receptor inhibitors because a potential upregulation of ACE2 would facilitate and worsen SARS-CoV-2 infection (Fang et al., 2020; Wan et al., 2020). According to a recent study, the lack of statistically validated data precluded an unambiguous statement on whether ACE inhibitors or ARBS improve or worsen the severity of COVID-19 patients (Iaccarino et al., 2020). However, a recent retrospective study on a large cohort of 18,472 patients tested for COVID-19 finds no association between ACEI or ARB use and positivity for the disease (Mehta et al., 2020). Several medical societies worldwide have expressed a strong recommendation to maintain ACEI and ARB medication in SARS-CoV-2 infected patients (see Tignanelli et al., 2020).

In the case of diabetes among COVID-19 patients, most of the studies find diabetes mellitus the second most frequent comorbidity, especially among the more severe cases requiring hospitalization (Fang et al., 2020; Huang et al., 2020; Zhang B. et al., 2020; Zhou F. et al., 2020); about 20% of the severe COVID-19 patients admitted to ICUs presented diabetes as comorbidity (Zhou F. et al., 2020). This is not surprising, given the association of the disease with infection and the susceptibility of diabetic patients to worse outcomes (Pearson-Stuttard et al., 2016). In fact diabetes type 2 is associated with a low grade chronic inflammation related to or induced by excessive adipose tissue in multiple organs of the economy (Iacobellis, 2020). According to this author the transmembrane enzyme DPP4 plays a major role in glucose and insulin metabolism, degrading incretins such as glucagon like peptide 1 (GLP-1) and glucose-dependent insulinotropic polypeptide, thus contributing to reduced insulin secretion and abnormal visceral adipose tissue metabolism. DPP4 also plays a role in immune regulation by activating T cells, and is purported to increase inflammation in diabetic patients (Iacobellis, 2020) In the case of COVID-19, diabetes has been suggested to modulate the host-viral interactions and contribute to ineffective host immune responses (Muniyappa and Gubbi, 2020). In an experimental model of ARDS, which is associated with high severity and mortality among COVID-19 patients, the DPP4 inhibitor sitagliptin decreased histological signs of lung injury by hindering the release of pro-inflammatory cytokines IL-1β, TNFα, and IL-6 (Kawasaki et al., 2018). Being a chronic inflammatory condition, diabetes is associated with multiple pathologies including vasculopathies (Klok et al., 2020) which are in turn closely related to the coagulopathies increasingly being observed in COVID-19 (Arachchillage and Laffan, 2020; Hussain et al., 2020; Klok et al., 2020; Mei and Hu, 2020; Shi et al., 2020; Wu et al., 2020).

The progressively mounting evidence on the involvement of inflammation and hyperreactivity of the immune system in COVID-19 refocuses attention on the anti-inflammatory action of the ACE2 arm in RAAS. The “dilemma,” as recently put by AlGhatrif et al. (2020), of whether to follow expert opinions and employ ACEIs/ARBs in cardiovascular patients or discontinue their use in those affected by COVID-19 is still alive. A dichotomy between the role of ACE2 expression in young (who are expected to have higher levels of the enzyme) or older adults (with age-dependent lower ACE2 levels and exacerbation of the angiotensin II pro-inflammatory arm) in COVID-19 outcomes is beginning to take shape (AlGhatrif et al., 2020; Bavishi et al., 2020). The latter authors hypothesize that SARS-CoV-2 binding to ACE2 acutely exaggerates the pro-inflammatory background.

In the short run, the current COVID-19 pandemic is posing challenges that require unprecedented speed to resolve, in many instances with insufficient evidence, as seems to be the case with hypertension, diabetes and other presumed co-morbidities and their pharmacological management. In the long run, some authors hypothesize that upon control of the current COVID-19 pandemic the SARS-CoV-2 will reemerge in its present or in mutated forms and become chronic (Patel and Verma, 2020). If this turns out to be the case, a deeper understanding of the RAAS system in relation to CoV infection will be important, as will the need to establish appropriate protocols for patients suffering cardiovascular diseases and diabetes, the most common non-communicable epidemic diseases.

## ACE2, The Main SARS-CoV-2 Host Cell Receptor

Non-endothelial cells in heart and testis and tubular epithelium of the kidney (Donoghue et al., 2000). Subsequent work screened and found its expression in oral and nasal mucosae and pulmonary alveolar and gastrointestinal epithelia, suggesting possible routes of viral entry (Hamming et al., 2004; Xu H. et al., 2020). An RNA-seq profiling study designed to explore the putative presence of ACE2 in the epithelial mucosa of the human oral cavity found highest expression in the tongue in comparison to the gingival or the rest of the buccal mucosa (Xu H. et al., 2020).

Pulmonary alveoli are lined by two types of epithelial cells or pneumocytes: type 1 pneumocytes (AT1) are non-replicating, large and relatively flat cells whose main function is to regulate the O_2_-Co_2_ exchange. The smaller AT2 cells are involved in the production and secretion of surfactant and a subpopulation of these can re-differentiate into AT1 and other cell types upon alveolar damage. SARS-CoV primarily targets ACE2 in ciliated bronchoalveolar epithelial cells and AT2 cells (Li et al., 2003; Qian et al., 2013) whereas MERS virus preferentially attacks AT2 cells (Rockx et al., 2020). The massive attack of AT2 cells by SARS-CoV-2 may significantly reduce their capacity to synthesize surfactant, a factor that combined with the lower surfactant-producing capacity in the elderly, may partly explain the severity of the pulmonary complications in older patients (Grasselli et al., 2020; Zhou F. et al., 2020). A recent immunocytochemical study has further narrowed down the focus and localized expression of ACE2 in the motile cilia of the respiratory tract epithelia, and demonstrated that factors such as patient demography, clinical presentation, and co-morbidities modify such expression (Lee et al., 2020). One such co-morbidity is smoking, which reduced mucociliary clearance by affecting the morphology of airway epithelial cilia (Leopold et al., 2009).

A recent graph-based bioinformatic data analysis corroborated that the highest, though not exclusive, expression of ACE2 was in a very small proportion (0.64%) of all human pulmonary cells. Of these, the vast majority (80%) corresponded to AT2 cells (Zhao et al., 2020). In a recent study of COVID-19 in an animal (macaque) model system, both AT1 and AT2 pneumocytes, but predominantly AT2, were infected as well as ciliated epithelial cells of nasal, bronchial, and bronchiolar mucosae with severity increasing from MERS to SARS to SARS-CoV-2 (Rockx et al., 2020). Interestingly, several other ACE2-related genes that facilitate viral reproduction and transmission are also highly expressed in the AT2 cells (Zhao et al., 2020). Using a novel functional viromics approach, the receptor binding domain of lineage B (beta)-CoVs can be divided into functionally distinct clades. ACE2 was found to be the entry receptor specific for clade 1 of such lineage. When tested with this new assay, SARC-CoV-2 incorporated into cells expressing ACE2, but not into other cell-surface receptors. Furthermore, several viruses exhibit compatibility with a still unknown receptor in human cells (Letko et al., 2020). ACE2 is developmentally regulated in mouse pulmonary epithelium (Wiener et al., 2007). The lack of manifest clinical symptomatology and/or severe forms of COVID-19 in children and adolescents below the age of 15 mentioned above could be related to the presence of an immature form of ACE2 in this population, or lower expression levels of the enzyme, as recently documented in a study of 305 individuals aged 4–60 showing a clearly lower *ACE2* gene expression in children <10 years old (Bunyavanich et al., 2020).

Not all ACE2 isoenzymes were born equal. Coding variants in different populations have recently been noted, and such variability may account for binding affinities. ACE2-K26R is more abundant in Ashkenazi Jews; this isoform has a lower electrostatic attraction for SARS-CoV-2. In contrast, the virus binds more to missense variants ACE-I468V, R219C, K341R, D206G, and G211R (in increasing order), corresponding to frequencies observed in East Asian, South Asian, African and African American, European, European and South Asian populations, respectively. The latter isoforms exhibit higher electrostatic attraction, dominated by van der Waals forces (Ali et al., 2020).

## Structures of CoV Surface Spike Protein S and Host Cell Receptor, ACE2

Development of drugs to tackle the current pandemic are urgently needed, and knowledge of the structure of the virus, the host cell receptor, and of the complex between the two are key to guide drug design and repurpose existing drugs. During the last few weeks there has been an explosion of high-resolution X-ray crystallographic and cryo-electron microscopy (cryo-EM) studies on the structure of the SARS-CoV-2 alone or in complex with ACE2. This would have been impossible to accomplish in such a short period were it not for the pioneer X-ray crystallography work started more than 15 years ago on the structure of SARS-CoV and MERS-CoV (Supekar et al., 2004; Li et al., 2005).

**Figure 1:**
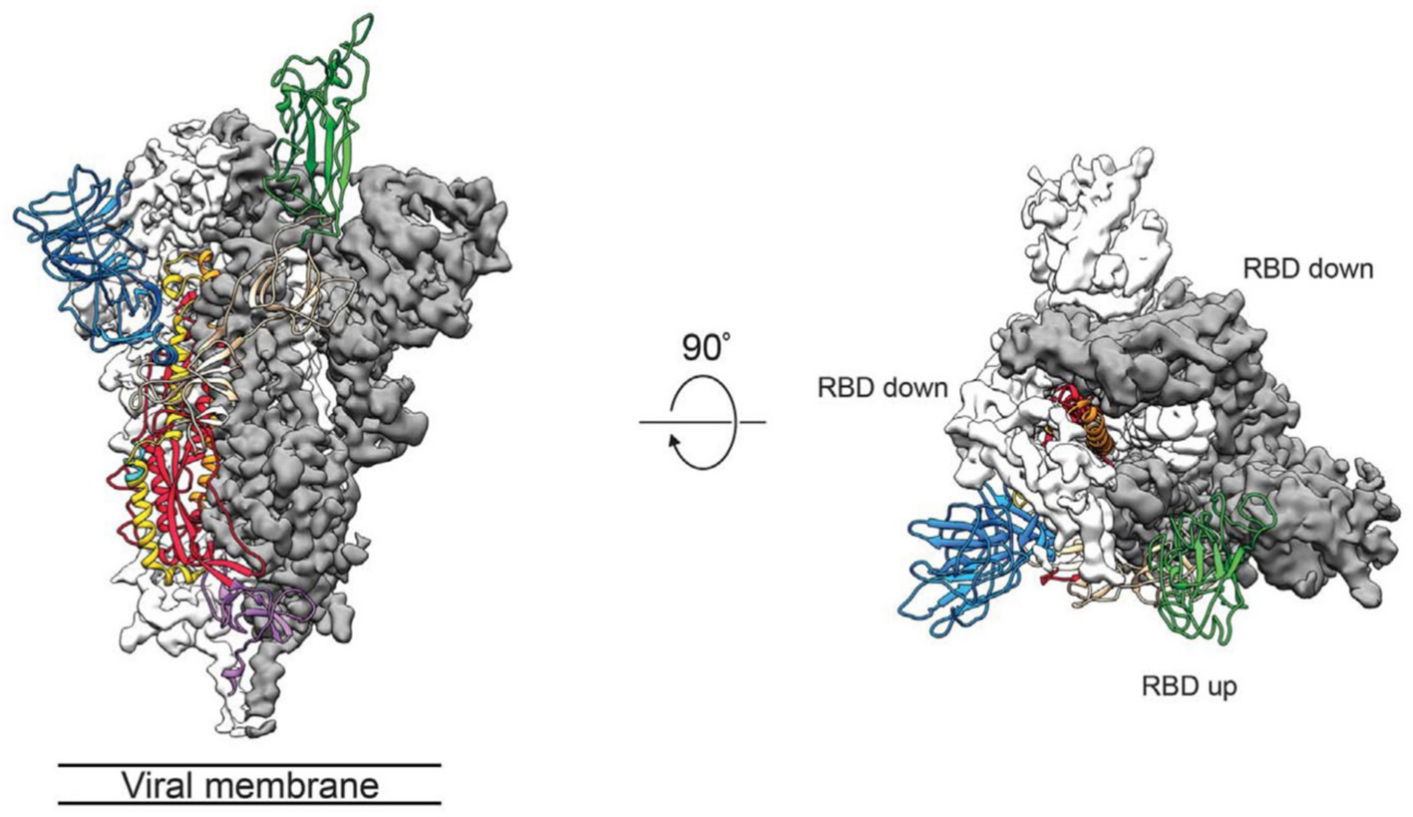
Side (left) and top (middle) views of the SARS-CoV-2 S protein prefusion structure with a single RBD in the “up” conformation obtained by cryo-EM. The two RBD down protomers are shown in either white or gray surface rendering; the RBD “up” protomer is shown in green ribbon rendering. From the cryo-EM study at 3.5 Å resolution of Wrapp et al. (2020) (PDB 6VSB), used with permission from Science AAAS.

**Figure 2:**
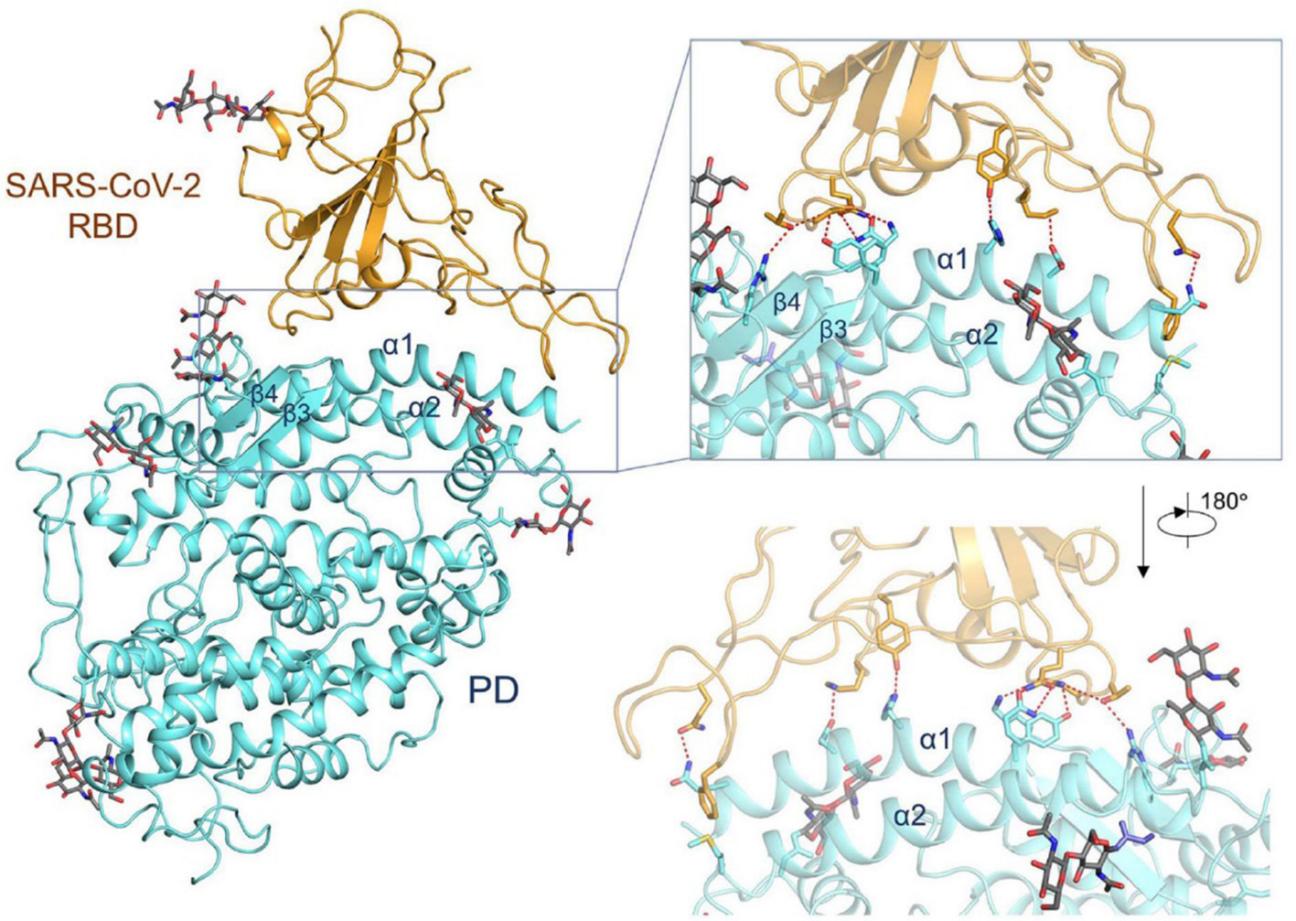
Interactions between the receptor-binding domain (RBD) of SARS-CoV-2 and its cell-surface receptor molecule, the enzyme ACE2. The latter (light blue-ribbon rendering) engages essentially a single linear motif, the α1-helix, to recognize the corresponding viral RBM (golden rendering), with contributions from the α2-helix, as can be appreciated in the inset. From the cryo-EM study at 2.9 Å resolution of the SARS-CoV-2 RBD in complex with ACE2 in the presence of the neutral amino acid transporter, B°AT1 (PDB 6VW1) (Yan et al., 2020), held under Creative Commons license. The contact area between the a loosely packed binding motif in the RBD and the two long helices of ACE2 is ~1,700 Å^2^ (De Sancho et al., 2020).

The group of McLellan has recently obtained the pre-fusion structure of the SARS-CoV-2 S protein at 3.5 Å resolution (Figure 1) and tested the putative binding of monoclonal antibodies against the RBD of SARS-CoV, without finding any apparent antibody cross-reactivity to SARS-CoV-2 (Wrapp et al., 2020). The structure is quite similar to that of the S protein from the human beta-CoV HKU1-CoV also obtained by cryo-EM (Kirchdoerfer et al., 2016). Another recent cryo-EM study has resolved the structure of the full-length human ACE2 with or without the RBD of the S1 spike protein of SARS-CoV-2, in the presence of a neutral amino acid transporter, B°AT1, which awards stability to the crystal structure, at a resolution of 2.9 Å (3.5 Å at the RBD) (Yan et al., 2020). This is the most detailed structure of ACE2 to date (Figure 2). Yet another cryo-EM contribution has recently determined the structure of the SARS-Cov-2 spike glycoprotein trimer and shown that it carries a furin cleavage site between the S1 and S2 subunits, a peculiarity of SARS-CoV and other CoVs (Wells, 2020). The S protein of SARS-CoV-2 contains 4 redundant Pro-Arg-Arg-Ala amino acid motifs which are not present in SARS-CoV. The proprotein convertase furin fulfills the function of detaching these motifs to enable the activation of the spike S protein and entry into the host cell (Shang et al., 2020). The existence of the furin mechanism overcomes the “default” state (mostly in the “down” conformation) of the SARS-CoV-2 spike RBD, which is inefficient for host cell binding.

One viral RBD is recognized by one extracellular peptidase domain of ACE2, in a single molecule-to-molecule fashion (Figure 2), in a manner akin to the recognition of SARS-CoV S1 protein by ACE2 (Li et al., 2005; Song et al., 2018; Hoffmann et al., 2020a; Wells, 2020; Yan et al., 2020).

The affinity of ACE2 for the SARS-CoV-2 S1 spike protein RBD has recently been reported to be similar (Correa Giron et al., 2020; Wells, 2020) or 10–20 times higher than that of SARS-CoV (Wrapp et al., 2020). *In silico* modeling of the SARS-CoV-2 protease involved in virion entry into cells has disclosed a high degree of flexibility of the protein, which not only involves the site where a known inhibitor binds, but also exposes other putative sites where enzyme blockers could bind (Wells, 2020). Another molecular dynamics study comparing the differences between SARS-CoV and SARS-CoV-2 modes of binding to ACE2 suggests that the RBD of the former virus has a stronger interaction with the complementary ACE2 site (Chen Y. et al., 2020).

All in all, the structural information that has become available in the last few years -and weeks- provides a still fragmentary but comprehensive picture of the virus-cell receptor complexes at atomic resolution with obvious implications for future structure-based drug and vaccine design. At the time of resubmitting this review (May 22, 2020) several vaccine candidates are being developed at an unprecedented pace using various strategies analyzed in Corey et al. (2020).

## Clinical Epidemiology and Risk Factors for COVID-19

Lower respiratory tract infection with fever, dry cough, and dyspnea are the most common, usually associated, presentations of COVID-19 (Huang et al., 2020), and of these dyspnea appears to be the most frequent predictor of the severe forms of the disease and ICU admission (Jain and Yuan, 2020). The majority of COVID-19 patients admitted to ICUs have been diagnosed and treated as severe cases of pneumonia (Arentz et al., 2020; Chen L. et al., 2020; Jain and Yuan, 2020; Wang D. et al., 2020; Zhou F. et al., 2020). Initial reports pointed to several comorbidities that constituted risk factors associated with severe forms of the disease; these included hypertension, diabetes, cardiovascular disease, older age, smoking, and chronic obstructive pulmonary disease (Wu and McGoogan, 2020). Asthma does not appear to be among the risk factors, as reported in the same study on a large cohort of 72,314 patients, of which 44,672 were confirmed COVID-19 cases (Wu and McGoogan, 2020). Other studies pointed to a peculiar age distribution, which unlike other viral diseases, largely spares children and adolescents below the age of 15 from the clinically manifest forms (del Rio and Malani, 2020; Li Q. et al., 2020; Wu and McGoogan, 2020; Young et al., 2020), accounting for <2% of the total cases of COVID-19 (Wu and McGoogan, 2020). New statistics indicate that children <18 years old represent <1.7% of all COVID-19 cases in the USA, 1% in the Netherlands and 2% in the UK (Viner and Whittaker, 2020). The authors discuss whether these figures “*reflect lower susceptibility of children versus adults, or similar infection rates but much higher proportions with asymptomatic disease*.” However, as the number of COVID-19 cases grow, so does the number of children affected with a severe form of the disease. Most of these children have been diagnosed with a multisystem inflammatory syndrome, in some cases leading to shock (Viner and Whittaker, 2020), a condition that has been interpreted as a delayed response to the viral infection. This presentation of the disease bares similarity to the Kawasaki disease, as recognized in an initial report from Italy referring to the COVID-19 affected children as presenting a Kawasaki-like disease (Verdoni et al., 2020). Kawasaki disease is a rare, acute and usually self-limiting vasculitis of the medium caliber vessels, which almost exclusively affects children (Viner and Whittaker, 2020). Half of the children in the Italian study required resuscitation (Verdoni et al., 2020); without treatment up to 25% of cases can present coronary artery aneurisms (Harahsheh et al., 2020), attesting to the severity of the syndrome. The disease has been termed pediatric inflammatory multisystem syndrome temporally associated with COVID-19 (PIMS-TS). The initial notion that severe forms of COVID-19 are associated only with adults, particularly elderly adults, potentially evolving to fatal outcomes, is therefore changing; children share this severe prognosis in a minority of cases.

## Perspectives and Possible Avenues for Drug Repurposing and Development

While much of what we need to know about the viral spread, contagiousness, clinical evolution, prognosis, impact of control measures for disease mitigation and other variables of the COVID-19 disease remains terra incognita, critical examination of the literature data shows that the considerable progress accomplished in some basic aspects of other CoVs like SARS-CoV or MERS, and more recently on SARS-CoV-2 and host receptor(s) offers hints at possible developments aimed at ameliorating disease progression and hopefully helping therapeutic approaches to materialize. More thorough analyses of physicochemical properties of anti-CoV drugs can be found e.g., in Liu et al. (2020).

### Precise Specification of Viral Cellular Targets Through Single-Cell Profiling and Other Novel Techniques

The organ and tissue distribution of viral receptors, as well as their expression levels, appear to be directly related to the choice of cellular targets and infection routes adopted by the viruses. An ACE2 RNA expression profile of normal human pulmonary tissue showed that ACE2 is highly expressed in AT2 human pulmonary cells, but that these constitute only a small percentage (0.64%) of the bronchoalveolar epithelial lining (Zhao et al., 2020). High expression is also found in the tongue (Xu H. et al., 2020); however, the oral cavity, with a reported surface area of 215 cm^2^ (Collins and Dawes, 1987), represents only a minute surface fraction compared to the upper respiratory tract and the total pulmonary alveolar area (118 ± 22 m^2^ and 91 ± 18 m^2^ in male and female individuals; Colebatch and Ng, 1992). The total areas of the mucosae appear to be the only quantitative variable available; we ignore receptor absolute numbers in each mucosa, their density, and binding affinities required to assign the relative probabilities of viral entry through each mucosa.

The technologies for conducting next-generation RNA sequencing at the single-cell level (scRNA-seq) are currently a reality. Such developments make it possible to undertake transcriptome-wide analysis of differential gene expression and differential splicing of mRNAs, and establish the tissue distribution of molecular constituents in individual cells (spatial transcriptomics, “spacialomics”) with unprecedented discriminative power that surpasses conventional antibody-based immunocytochemistry (for a review see Stark et al., 2019). Another promising technique of recent implementation is “TooManyCells,” a suite of graph-based algorithms that can be applied to partition RNA-seq data to resolve and visualize clusters of cells and establish their relationships in an unbiased manner (Schwartz et al., 2020). With this technique only relatively rare sets of cells, representing a mere 0.5% of the population, could be resolved. Through these approaches, host-cell selectivity of viral receptors could be narrowed down to the level of cell populations, and subpopulations within a tissue or organ with great precision, with inherent prophylactic and therapeutic implications. Differences in the expression of ACE2 missense mutants in different ethnicities were reported (Ali et al., 2020). Such heterogeneity could also be reflected at the tissue and cell levels in a given individual, explaining the variabilities in risk, susceptibility and vulnerability of certain organs to become targets of the disease. Data collected from more than 4 million human cells by a large consortium of scientists in the Human Cell Atlas project has recently shown through RNA-seq analysis that SARS-CoV-2 can infect more cell types than previously known (Muus et al., 2020). It also made apparent the occurrence of cells co-expressing both ACE2 and the accessory proteases that identify specific subsets of respiratory epithelial cells as putative targets of viral infection in the nasal passages, airways, and alveoli.

A recent immunocytochemical study has further narrowed down the focus and localized expression of ACE2 in the motile cilia of the respiratory tract epithelia, and demonstrated that factors such as patient demography, clinical presentation, co-morbidities modify such expression (Lee et al., 2020).

### Exploiting Our Current Knowledge of SARS-CoV-2 and Other CoVs to Inhibit Virus Binding to Receptors

#### ACE2-RBD Inhibitors

Work on the structures of the S spike protein of CoVs and ACE2 and their complexes has produced important information and ideas not only on the relevant epitopes for vaccine design (Li et al., 2003; Supekar et al., 2004; Imai et al., 2005; Struck et al., 2012; Song et al., 2018) but also for interventions ranging from application of recombinant ACE2 as protection against severe acute lung failure (Imai et al., 2005) to the use of ACE2 inhibitors in CoV diseases (Imai et al., 2005; Struck et al., 2012; Li, 2013; Song et al., 2018). A number of ACE2 peptide inhibitors with nanomolar affinity and a non-peptide blocker with sub-nanomolar affinity, MLN-4760, have been tested on the soluble form of ACE2 (Warner et al., 2004). Current studies addressed at testing inhibitors of viral infection *in vitro* have reported promising results with cepharanthine, selamectin, and mefloquine hydrochloride, three drugs that appear to hinder cytopathic effects of the GX_P2V virus, which is 92% homologous to SARS-CoV-2 (Fan et al., 2020); no clinical studies have been reported.

#### The TMPRSS2 Protease

**Figure 3:**
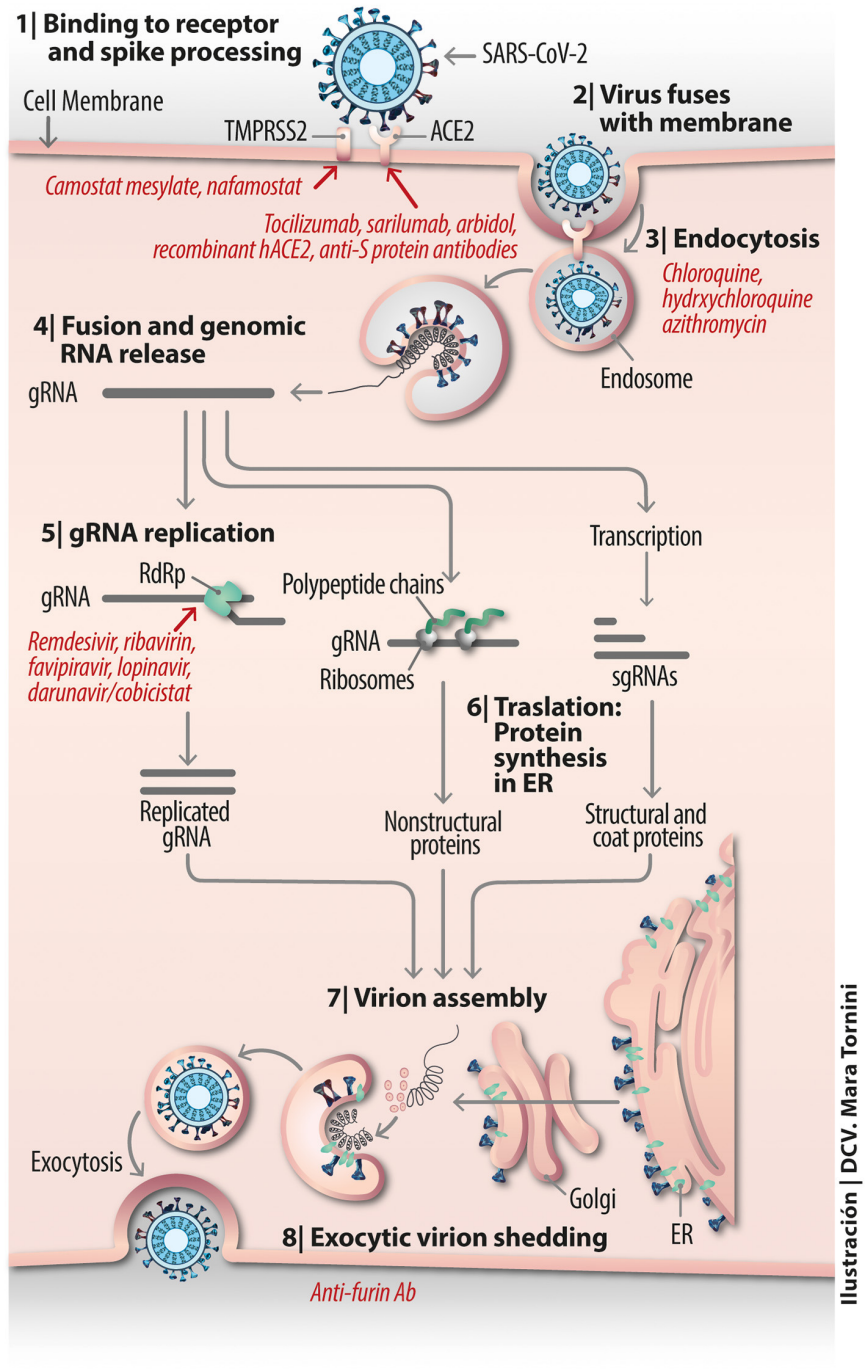
Vital cycle of SARS-CoV-2 from host cell receptor binding to exocytic virion shedding.

The membrane-bound protease TMPRSS2 presumably serves the role of co-receptor for SARS-CoV-2 engagement with ACE2 (Matsuyama et al., 2020). It has been shown to be blocked by camostat mesylate (Hoffmann et al., 2020a), a serine protease inhibitor previously found to inhibit Ebola and SARS-CoV entry (Zhou et al., 2015). The drug has been clinically proven in oncological therapy and treatment of pancreatitis in Japan (Kawase et al., 2012) and shown efficacy *in vitro* in combination with cathepsin inhibitors in SARS-CoV infected human HeLa cells expressing ACE2 and TMPRSS2. A possible line of action in drug development is to use this type of compounds as a template on which to optimize the design of more selective and/or more effective active-site or allosteric site inhibitor drugs. Nafamostat mesylate, another serine protease inhibitor that has been used in Japan for more than 30 years for pancreatitis and as an anticoagulant of perfused blood known to inhibit TMPRSS2-mediated host-cell entry of MERS-CoV (Figure 3) and presumptively of SARS-CoV-2 (Iwata-Yoshikawa et al., 2019), has been recently employed alone, to block activation of SARS-CoV-2 (Hoffmann et al., 2020b), or in combination with heparin as an anti-enhanced fibrinolysis therapy for the coagulopathies observed in severe cases of COVID-19 (Asakura and Ogawa, 2020).

### Inhibiting Virus Endocytic Internalization and Intracellular Trafficking

To enter the cells, many viruses appropriate canonical endocytic pathways used by cells under physiological conditions. One of the earliest mechanisms of CoV endocytosis studied is that of mouse hepatitis virus MHV-4; infection occurs via endocytic and non-endocytic mechanisms (Nash and Buchmeier, 1997). Viral endocytic internalization operates via a clathrin-dependent, caveolin- and EPS-15-independent mechanism (Pu and Zhang, 2008). Drugs that inhibit the clathrin- and dynamin-dependent endocytic pathway impede dengue virus infection of mononuclear phagocytic cells (Carro et al., 2018). Temporary inhibition of endocytosis with the anti-emetic/anti-psychotropic drug prochlorperazine is deemed to be potentially safe in humans (Chew et al., 2020). The SARS-CoV S protein is digested by cathepsin L or B in the lumen of the endocytic compartment, and on this basis the use of protease inhibitors has been explored as potential broad-spectrum anti-CoV agents, including SARS-CoV (Zhou et al., 2015). Expression of exogenous cathepsin L significantly enhances SARS-CoV but not HNL63-CoV infection via ACE2 (Huang et al., 2006). This exemplifies how two CoVs that target a common receptor molecule infect the cells using different mechanisms.

Experimental demonstration that SARS-CoV-2 enters HEK-293 cells engineered to express human ACE2 *in vitro* (HEK-293/hACE2) via an endocytic mechanism has become available (Ou et al., 2020). Cathepsin plays a critical role in this process. Viral endocytic mechanisms have gained recent attention partly because there are drugs available to inhibit specific internalization pathways. Endosomal maturation is accompanied by a drop in the endosomal pH. The relatively simple organic anti-malaria compounds chloroquine and hydroxychloroquine (see Figure 3 and recent review in Touret and de Lamballerie, 2020), used since before mid-twentieth century, is known to moderately raise the pH of endosomes and/or lysosomes, hindering viral fusion, uncoating and further processing in the endosomal lumen and lysosome.

Additional mechanisms beyond inhibition of endocytic internalization are suggested by biotechnological studies on nanoparticle uptake by macrophages (Hu et al., 2020) The possible prophylactic or therapeutic effects of chloroquine and its derivative hydroxychloroquine, also employed for autoimmune diseases (Colson et al., 2020), lupus and rheumatoid arthritis, is currently being subjected to clinical trials for COVID-19 in China and France (Devaux et al., 2020); critically validated results are still being awaited within the context of the SOLIDARITY program launched by the World Health Organization (see Table 1). Both drugs have a rather narrow margin between therapeutic and toxic effects, some of them lethal (Touret and de Lamballerie, 2020). It is contended that chloroquine and derivatives not only affect the endocytic mechanisms of viral entry but may also interfere with the SARS-CoV-2 replication cycle (Devaux et al., 2020). A recent retrospective multi-center study on a large cohort of randomly chosen 1,438 patients treated with hydroxychloroquine alone or in combination with the antibiotic azithromycin in 25 hospitals (representing 88.2% of all hospitalized COVID-19 patients in New York) showed no significantly lower mortality but more frequent ventricular arrhythmias and cardiac arrest in patients medicated with the drug combination (Rosenberg et al., 2020) (Table 1).

**Table 1:** Some examples of current clinical trials with repurposed drugs.

**Drug trial**	**Outcome**	**Observations**	**References**
Lopinavir/ritonavir	No benefit was observed with lopinavir-ritonavir treatment beyond standard care.	Patients (*n =* 199) with similar viral RNA load and clinical status. Median of 13 days from onset of disease to randomization	Cao et al., 2020
Lopinavir/ritonavir	Pending secondary outcome analyses, the study concludes suggesting substantial diminution of overall mortality (19 vs. 25 in standard care group), risk of respiratory failure (13% vs. 27%)	Contradicts conclusions of Cao et al. (2020). Patients (*n =* n/a) in this trial underwent randomization a median of 13 days after disease onset. Initiating therapy earlier may be more effective, since systemic hyperinflammation rather than viral pathogenicity dominates later stages	Dalerba et al., 2020
Darunavir/cobicistat plus hydroxychloroquine	A “post-exposure prophylaxis (PEP)” trial aimed at treatment of COVID-19 cases with the HIV drug combination darunavir/cobicistat plus hydroxychloroquine treatment and chemoprophylaxis of household contacts having spent >15 min with infected patients. Results pending	To evaluate the efficacy of the “test and treat” strategy of infected patients and prophylactic chloroquine treatment to all contacts (Patients recruited *n =* 1,000). Decentralized COVID-19 testing and starting chloroquine treatment immediately in all who are found to be infected. Control group receives no medication	Fundacio Lluita Contra la SIDA in collaboration with Department of Health, Generalitat de Catalunya, German Trias I Pujol University Hospital and private laboratories, Barcelona, Spain. Press release U.S. National Library of Medicine (ClinicalTrials.gov)
Hydroxychloroquine	No difference, but sampling may not have been adequate	Patients (*n =* 150) with too late (28 day) viral clearance and randomized too late in the course of the disease	Tang et al., 2020
Chloroquine/ Hydroxychloroquine	Chloroquine or placebo in Asia, hydroxychloroquine in Africa. Expected outcome: After 5 months follow up determine clinical conditions of prohylactic/therapeutic approach. Estimated commencement: April 2020; end: April 2021	Participants (expected = 40,000) of a double-blind randomized, placebo-controlled trial to be conducted in healthcare settings or COVID-19 proven or suspected individuals	U.S. National Library of Medicine press release (ClinicalTrials.gov)
Hydroxyhloroquine alone or in combination with azytromycin	No significant lower mortality; more frequent cardiac arrest in patients treated with the drug combination	Patients (*n =* 1,438) from 25 hospitals representing 88.2% of all hospitalized COVID-19 individuals in New York	Rosenberg et al., 2020
Hydroxychloroquine	A phase 2/3 trial to test kinetics of hydroxychloroquine medication in a blind, randomized fashion. Start date: April 17, 2020. Completion due: July 1, 2020	Patients (*n =* 58) medicated with hydroxychloroquine to test whether medication will decrease the amount of virus (as measured by PCR), 7 days after initiation of therapy compared to control randomized patients receiving placebo	U.S. National Library of Medicine press release (ClinicalTrials.gov)
Hydroxychloroquine or chloroquine, w/without macrolide	Either drug alone or in combination with a macrolide increased the risk of *de-novo* ventricular arrhythmia. Trial started 20 December, 2019 and ended April 14, 2020	Multi-center study including 96,032 COVID-19 hospitalized patients (671 hospitals in six continents) medicated with chloroquine alone, hydroxychloroquine alone, or either drug in combination with a macrolide	Mehta et al., 2020
Lopinavir/ritonavir vs. umifenovir vs. control	No difference for primary or secondary outcomes	Patients (*n =* 86) in an open-label trial randomized 2:2:1 The primary endpoint set at time of negativity of RT-PCR	Li Q. et al., 2020
Hydroxychloroquine	Prospective clinical study in the prevention of COVID-19 among healthcare personnel after high exposure to SARS-CoV-2. Start date: April 3, 2020. Estimated completion date: July 30, 2020	Participants (*n =* 336) non-randomized, parallel assignment; 50% subjected to oral hydroxychloroquine for a total of 7 weeks. Baylor University Medical Center, Dallas, Texas	U.S. National Library of Medicine press release (ClinicalTrials.gov)
Remdesivir	Non-statistically significant tendency suggesting less mortality rate in a subset cohort starting medication within 10 days of symptom onset	Patients (*n =* 236) assigned to remdesivir or placebo groups. Randomization according to type of non-assisted/supported respiration. Many patients received steroids	Wang et al., 2020 (Retracted by the journal The Lancet)
Remdesivir	Remdesivir-treated patients showed accelerated (31%, median 11 days) recovery relative to placebo group (median 15 days, *p < * 0.001). Results also suggest survival benefit (mortality rate 8% vs. 11.6 in placebo group). Most beneficial for severe cases requiring oxygen supply	Patients (*n =* 1,063) were hospitalized COVID-19 patients from 10 countries recruited by NIH National Institute of Allergy and Infectious Diseases (NIAID) for a randomized, controlled clinical trial named Adaptive COVID-19 Treatment Trial (ACTT)	NIH press releases of April 29 and May 22, 2020 and Beigel et al., 2020
Remdesivir	No differences were observed in the improvement of severe COVID-19 patients who received remdesivir for 5 or 10 days. 10% of the patient developed acute respiratory failure	Hospitalized patients (*n =* 397) underwent a 5 or 10 day course randomized treatment. A phase 3 trial named SIMPLE by commercial firm (Gilead Sciences, Inc.). No placebo group. Study will be extended to enroll 5,600 patients at 180 locations	Press release Gilead Sci. Inc.
Tocilizumab	To evaluate the drug tocilizumab, a monoclonal antibody that blocks interleukin-6 receptor. Primary outcome was for either need for mechanical ventilation or death. Preliminary data indicate “tocilizumab improves significantly clinical outcomes”	Patients (*n =* 129) with moderate to severe COVID-19. Open-label, randomized, multi-center, controlled trial conducted in Paris hospitals to evaluate efficacy and tolerance of various immune modulators. No placebo group (50% patients standard of care alone). Final report under peer review	Press release of CORINMUNO-19 platform, Paris, 27 April, 2020
Tocilizumab	To establish proof-of-concept that the drug tocilizumab, a monoclonal antibody that blocks interleukin-6 receptor is effective in decreasing clinical signs, symptoms, and laboratory evidence of COVID-19 pneumonitis. Begins April4, 2020; ends July, 2020	Patients (*n =* 50), hospitalized, non-critically ill with COVID-19, showing clinical risk factor(s) for decompensation, ICU utilization, or death. A second group of patients without risk factor for decompensation. Sponsor: Univ. of Chicago	U.S. National Library of Medicine press release (ClinicalTrials.gov)
Tocilizumab	A phase 2 trial on patients affected by severe multifocal interstitial pneumonia correlated to SARS-CoV2 infection. Start date: March 12, 2020. Estimated completion: May, 2020	Patients (*n =* 38) to test the hypothesis that an anti-IL-6 receptor drug calms the virus-induced cytokine storm, blocking deterioration of lung function or even promoting a rapid improvement of clinical conditions	U.S. National Library of Medicine press release (ClinicalTrials.gov)
Sarilumab	Sarulimab, a human IgG1 monoclonal antibody that binds specifically to both soluble and membrane bound IL-6 receptor, did not improve conditions of severe COVID-19 group of patients; the group did not progress to phase 3. New enrollment will only contemplate critically-ill patients	Patients (*n =* 457) underwent a phase 2/3 commercial-sponsored trial (Regeneron-Sanofi U.S.). The monitoring committee recommended continuing the trial of sarilumab (Kevzara) only with the critically-ill group of patients, discontinuing it with the severe group of patients	Press release of Regeneron (Tarrytown, N.J.) and Sanofi (Paris) on April 27, 2020
Sarulimab	Phase 2/3 trial to evaluate therapeutic effect and tolerance of Sarilumab. Commencement: March 2020; expected completion by March 2011	Patients (*n =* 239) from Paris Hospitals. Bayesian open labeled randomized clinical trial in patients with moderate, severe pneumonia or critical pneumonia associated with COVID-19	U.S. National Library of Medicine press release (ClinicalTrials.gov)
Ivermectin	A pilot, proof-of-concept trial. Commencement date: May 11, 2020. Completion date: June 30, 2020	Patients (*n =* 45) from University of Salta, Garrahan Pediatric Hospital, Buenos Aires, Argentina. Based on *in-vitro* studies indicating suppression of SARS-CoV-2 replication, the trial will evaluate clinical applicability of the drug in a randomized study at early stages of COVID-19	U.S. National Library of Medicine press release (ClinicalTrials.gov)
Nitazoxanide/ribavirin and ivermectin	A pilot, proof-of-concept trial to establish rate and time of viral clearance in subjects receiving the combination of three drugs. Commencement date: May, 2020. Completion date: May 2022	Patients (*n =* 100) with COVID-19 will receive the combination of nitazoxanide, ribavirin and ivermectin vs. a control group without medication for a duration of seven days in a randomized, sequential scheme	U.S. National Library of Medicine press release (ClinicalTrials.gov)
Telmisartan	The drug telmisartan will be assessed in a pilot study due to commence on May 15, 2020 and with an estimated conclusion in October 1, 2020	Patients (*n =* 400) from Hospital de Clínicas José de San Martín, Faculty of Medicine, University of Buenos Aires, will receive telmisartan twice daily in an open-labeled, parallel assignment randomized trial	U.S. National Library of Medicine press release (ClinicalTrials.gov)
Prazosin	The α-blocker prazosin will be tested to evaluate whether it reduces ICU admission rate or need of assisted ventilation among COVID-19 severe patients. If test proves successful, a follow-up is planned with COVID-19 positive individuals who are not yet hospitalized	COVID-19 patients (*n =* n/a) aged 45–85 at John Hopkins Hospital, Baltimore, will receive gradually increasing doses of the α-blocker prazosin (Minipress) for 6 days and compared with a control group of patients receiving “standard” treatment	Vogelstein et al., 2020

There is an additional component in the virion infection process: the cytoskeleton. Entry of the porcine hemagglutinating encephalomyelitis virus, a member of the CoV family, into N2 cells is facilitated by rearrangement of the cytoskeleton via the α5β1-FAK/cofilin/Rac1/cell division cycle Cdc42 pathway (Lv et al., 2019). Unfortunately, the ubiquity of the cytoskeleton precludes therapeutic interventions.

### ACE and ACE2 in the Context of RAAS: New Strategic Approaches

In addition to contemplating ACE2 as the molecule targeted by SARS-CoV-2, it is important to envisage this enzyme as a key target of therapeutic interventions based on its role in pulmonary inflammatory pathologies. Indeed, the ACE2/Ang (1–7)/Mas receptor pathway is a potential therapeutic target worth exploring for ameliorating allergic inflammation of the respiratory tract, respiratory airway remodeling, and airway hyperresponsiveness. Acute respiratory failure with bilateral infiltrates and hypoxemia, without hydrostatic pulmonary edema, configures a potentially severe nosological entity eventually leading to acute respiratory distress syndrome, an inflammatory condition involving increased vascular permeability and hypoxemic respiratory failure (Bellani et al., 2016). The syndrome appears to be one of the most common complications of COVID-19. The severity of pneumonia and acute respiratory failure is positively correlated with age-dependent disequilibria of the ACE/ACE2 ratio (Schouten et al., 2016; Tan et al., 2018). Furthermore, reduced ACE2 activity has been observed in experimentally-induced acute respiratory distress syndrome. Therapeutic intervention with a protease-resistant form of angiotensin (1–7) improved all symptoms, indicating the possible association of angiotensin (1–7) deficiency with the syndrome (Wösten-van Asperen et al., 2011).

As more is learned about the clinical aspects of COVID-19, it is recognized that a subset of these patients, more often those suffering severe forms of the disease, develop dysregulated systemic hyperinflammatory reactions also known as macrophage activation syndrome, cytokine release syndrome or “cytokine storm,” consisting in the over-production of pro-inflammatory mediator molecules, predominantly cytokines. These include interleukin (IL)-6, IL-2R, IL-8, tumor necrosis factor-α, and granulocyte-colony stimulating factor (2, 4–8) (Moore and June, 2020; Zhang B. et al., 2020). The cytokine release in response to the SARS-CoV infection had already been observed with alveolar cells in culture infected with this CoV (Qian et al., 2013). Among the possible strategies to address this syndrome is to interfere with the “master player” interleukin-6 (IL-6)/IL-6 receptor/gp130 axis (Uciechowski and Dempke, 2020) employing e.g., IL-6 receptor inhibitors (Kang et al., 2019). Corticosteroids are in general not recommended (Sanders et al., 2020). Tocilizumab and sarilumab, monoclonal antibodies targeting the IL-6 receptor, and siltuximab, a chimeric antibody targeting IL-6, are currently being investigated for the treatment of patients with COVID-19 and ARDS complications; the potential of tocilizumab to curb the cytokine storm was reported in a retrospective study of a small number of patients (*n* = 21) in China with severe/critical COVID-19 who underwent treatment with the anti-IL-6 monoclonal antibody tocilizumab. Patients showed important reductions in morbidity and need for mechanical ventilation (Xu et al., 2020a). The antibody has now entered into various clinical trials (examples in Table 1; additional clinical trials listed in Konig et al. (2020) and Clinical.Trials.gov). The cost and potential adverse reactions may restrict the use of this approach to patients in developed countries according to Konig et al. (2020).

A recent retrospective meta-analysis of a large number of patients with acute respiratory distress or pneumonia (*n* = 13,125 and *n* = 108,956, respectively) from all causes led to the inference that those patients who were taking α-blockers had a lower risk of requiring ventilation (by 35 and 16%, respectively) and a reduced risk of requiring assisted ventilation and dying (by 56 and 20%, respectively), compared to non-users of α-blockers (Vogelstein et al., 2020). Based on this information and previous knowledge that catecholamines enhance pro-inflammatory reactions through a self-amplifying loop mediated by α1-adrenergic receptors that increase the production and release of IL-6 and other cytokines, and the amelioration of these phenomena by pharmacological catecholamine blockade in animal models (Staedtke et al., 2018), a clinical trial testing prazosin, a post-synaptic α1-adrenergic receptor competitive antagonist is underway (Table 1).

### The SARS-CoV-2 and Its Endogenous Proteases

Attacking the virus itself is another attractive possibility. Any of the viral proteins and the mRNA are possible targets, especially using drugs already developed for other viruses or modifications thereof. Among such already existing drugs are the inhibitors of the endogenous CoV proteases active downstream of infection for the biosynthesis of polyproteins translated from the viral RNA. This constitutes an important avenue of research since these endogenous proteases are required for the processing of two viral polyproteins, pp1a and pp1ab, into the 16 non-structural proteins involved in the production of subgenomic RNAs that encode, in turn, no less than the 4 structural proteins of SARS-CoV-2 (Dai et al., 2020). M^PRO^, also termed 3CL^PRO^, is the key enzyme needed for the cleavage of the two polyproteins in SARS-CoV. Forty unsymmetrical aromatic disulfide compounds have been synthesized and tested *in vitro* and found to be reversible competitive inhibitors of M^PRO^ activity, blocking viral replication. *In silico* docking was used to validate the *in vitro* assays (Wang et al., 2017). In SARS-CoV-2 an important endogenous protease is the papain-like protease (PL^PRO^), whose atomic structure is still not known. Based on available crystallographic data of the PL^PRO^ from SARS-CoV and MERS-CoV, a recent homology modeling study found that the PL^PRO^ of SARS-CoV-2 is 97% homologous to that from a bat CoV, 80% to SARS-CoV and only 29% homologous to MERS-CoV (Stoermer, 2020).

**Figure 4:**
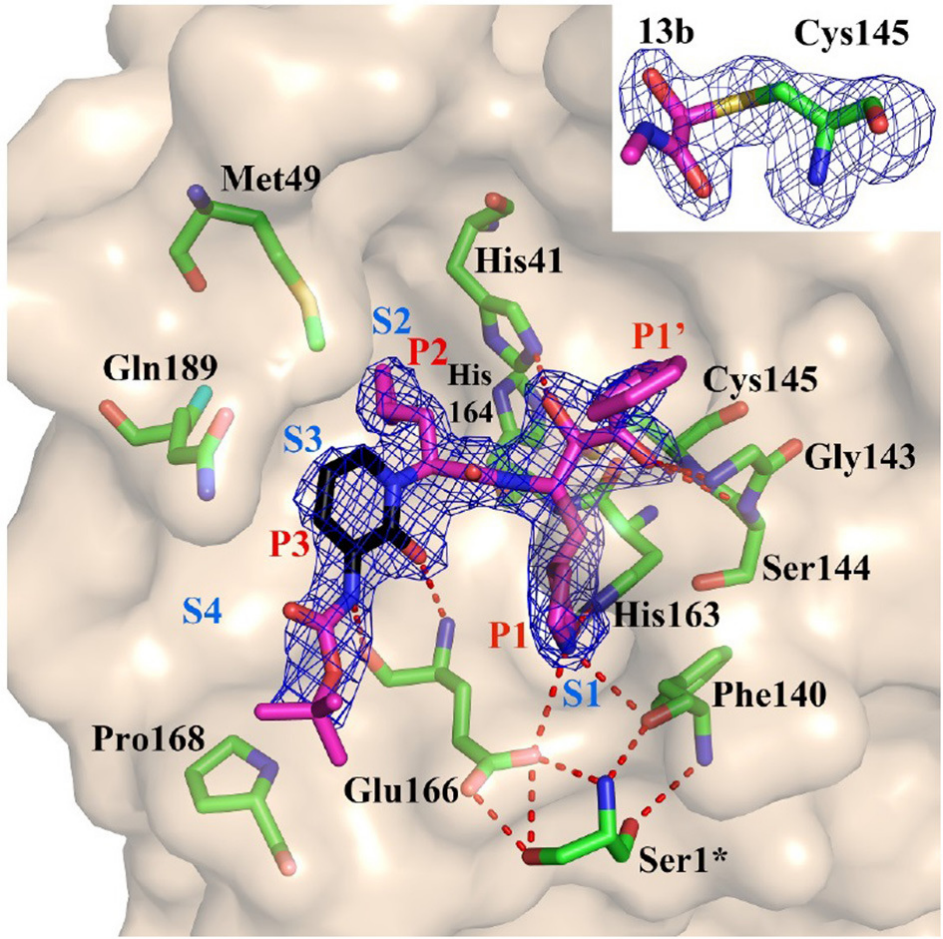
Substrate-binding cleft of M^PRO^, the endogenous protease in SARS-CoV-2, with compound 13b, a peptidomimetic α-ketoamide inhibitor of the viral enzyme. Fo-Fc density is shown for the inhibitor. Atom color rendering: magenta, carbon (except in the pyridone ring, which is black), red, oxygen; blue, nitrogen, and yellow, sulfur. Light-blue symbols S1, S2, S3, S4 indicate the canonical binding pockets for moieties P1, P2, P3, P4 (red symbols) inhibitor. Red dashed lines represent H-bonds. *Inset*: Thiohemiketal resulting from the nucleophilic attack of the catalytic Cys residue on the α-carbon of the inhibitor in its Fo-Fc density (contoured at 3σ). From Zhang L. et al. (2020), held under Creative Commons license.

The recent availability of the co-crystal of M^PRO^ from SARS-CoV-2 with an α-ketoamide inhibitor (Zhang L. et al., 2020) (Figure 4) and two structure-based design additional inhibitors, 11a and 11b (Dai et al., 2020) provides a structural basis on which to validate the *in silico* calculations and develop new virion entry blockers. A virtual screening of more than 3,000 compounds approved by the American Federal Drug Administration recently focused on the M^PRO^ of SARC-CoV-2 (Contini, 2020). Protease inhibitors previously or currently used in HIV retroviral therapy, like lopinavir, indinavir, and atazanavir were selected as potential candidates applicable to COVID-19 (Contini, 2020; Wang, 2020). The lopinavir +ritonavir combination has been used an HIV protease inhibitor, but a randomized, controlled study of 199 confirmed COVID-19 patients reported no benefits of dual drug administration, which was therefore interrupted (Cao et al., 2020) (see Table 1). Ritonavir inhibits the metabolizing enzyme cytochrome P450 3A, increasing the half-life of lopinavir. In another study the same FDA database was explored using free energy calculations, and dypyradamol was selected as the most promising drug, which according to the authors is undergoing clinical trials (Li Z. et al., 2020). Using the crystal structure of the viral proteases as a template, repurposing database screening combined with thermodynamics of ligand binding have highlighted the drug carfilzomib, an approved anti-cancer drug, as the best potential candidate to inhibit SARS-CoV-2 infection at the level of the proteasome, with a free energy of binding of −13.8 kcal mol^−1^. Antibiotics like ervacycline or streptomycin were also singled out (Wang, 2020). An important feature of blocking this viral protease, unlike other actors in the viral life cycle, is that no enzyme with such cleavage specificity is found in humans, thus minimizing the potential toxicity of inhibitors in therapeutic approaches to COVID-19.

In SARS-type CoVs, the non-structural protein 16 (nsp16) methylates the 5'-end of virally encoded 28 mRNAs to mimic cellular mRNAs, thus protecting the virus from host innate immune restriction. The 1.8 Å structure of a ternary complex of full-length nsp16 and nsp10 of SARS-CoV-2 in the presence of cognate RNA substrate and a methyl donor, S-adenosyl methionine, provide mechanistic information on viral mRNA cap (Viswanathan et al., 2020).

A proteomic and translational *in vitro* analysis has explored metabolic pathways followed -and altered- by SARS-CoV-2 when infecting cells, including translation, glycolysis, splicing, and nucleotide synthesis, identifying several small drugs inhibiting viral replication in cells in a temporal-dependent manner (Bojkova et al., 2020).

The diversity of candidate drugs resulting from “repurposing” database analyses, in some cases stemming from the same database, calls for alternative refining techniques. Deep learning is increasingly gaining momentum in protein structure prediction (Wardah et al., 2019; Senior et al., 2020; Singh, 2020). Deep learning strategies have also impacted on the field of drug design (Dana et al., 2018). Such strategies constitute a viable methodology which should be exploited to screen data banks of small drugs docking on cell host and viral proteases, SARS-CoV-2 surface proteins and other putative targets on the basis of structural and thermodynamic parameters, find new suitable small drug inhibitors, and explore *combinations of two or more drugs acting synergistically on different targets*.

### The SARS-CoV-2 RNA Polymerase

SARS-CoV-2 replicates its genome and transcribes its genes using an RNA-dependent RNA polymerase (RdRp), also called nsp12. The first-generation anti-HIV drugs aimed at the replicase enzymatic machinery responsible for RNA replication are the nucleotide analog drugs like remdesivir, a broad-spectrum inhibitor of RdRp originally designed for the Ebola virus and congeners, and whose effectiveness against COVID-19 is currently being investigated. Tested on a Rhesus macaque model of COVID-19, when applied early in the course of the experimentally-induced disease, the drug was effective in preventing the progress of the illness but did not inhibit virus shedding by the infected animals (Williamson et al., 2020). The anti-hepatitis C antiviral drug ribavirin is another of the potential drugs listed as candidates by the World Health Organization, but 26 out of 30 studies reviewed in a recent meta-analysis indicate inconclusive results and liver and hematologic toxicity with this drug in COVID-19 patients (Sanders et al., 2020).

Very recent cryo-EM studies have determined the structure of RdRp. The first, produced by the group of Xu and coworkers in Shanghai, solved the structure of the apo form of RdRp alone at 2.8 Å resolution, and in complex with a 50-base template-primer RNA plus the viral inhibitor remdesivir, at 2.5 Å resolution (Yin et al., 2020). From the structure of the complex these authors concluded that remdesivir works by acting as an immediate RNA chain terminator and suggested a rational template for future drug design. A second study at the Max-Planck-Institute for Biophysical Chemistry in Göttingen rendered the structures of RdRp (nsp12) together with the non-structural proteins nsp7 and nsp8 over two turns of the RNA template-product duplex (Hillen et al., 2020). These authors indicated that the work of Xe and coworkers contradicted biochemical evidence showing that remdesivir causes delayed chain termination after addition of more nucleotides. Aside from the differences in interpretation, the two studies coincide in their core structural findings, providing new insights into the mechanisms utilized by antivirals such as remdesivir, and in the design of better inhibitors of the viral RNA polymerase. A third study, also using cryo-EM at 2.9 Å resolution, solved the structure of RdRp in complex with cofactors nsp7 and nsp8, with inferences from parallel modeling studies on the possible use of favipiravir, given its similarity to remdesivir (Beigel et al., 2020; Gao et al., 2020) (see Table 1 for clinical trial). The therapeutic possibilities are thus not limited to using remdesivir only, but extend the RNA polymerase inhibitor strategy to a whole family of compounds including ribavirin, remdesivir, sofosbuvir, galidesivir, and tenofovir as well as other compounds like the guanosine derivative IDX-184, setrobuvir, and YAK, which have FDA approval in the U.S.A. (Elfiky, 2020).

### Lipid Metabolism in CoV-Infected Cells

Plus-strand RNA virus replication, as with CoVs, sequesters the lipid metabolic machinery in the infected cell, hijacking the enzymes normally involved in lipid synthesis and processing. Structurally this results in the formation of double-membrane vesicles and other abnormal membrane structures generically referred to as replicative organelles. These are the platforms for virion replication and transcription and virion morphogenesis. An inhibitor of the cytosolic phospholipase A_2_α, pyrrolidine-2, was found to reduce the formation of double-membrane vesicles in cells infected with the related human H229E CoV, impairing virion replication (Müller et al., 2018). The drug also diminished the formation of lyso-phospholipids, the products of phospholipase A_2_α enzymatic activity that are essential for CoV replication. It would be worth exploring the lipidomics of SARS-CoV-2 to investigate whether the virus possesses any singularity in terms of composition which can be exploited in its control.

A recent retrospective analysis of 861 patients with clinically classified mild, moderate, severe or critical (the latter with respiratory failure, shock or organ failure) COVID-19 analyzed the possible association with plasma cholesterol and HDL on morbidity and prognosis (Wei et al., 2020). The levels of total cholesterol and HDL were found to be inversely correlated with the severity of the disease, and severely ill patients with high HDL had a better prognosis. Survivors also had higher cholesterol and HDL levels. Furthermore, pretreatment with ITX 5061, a potent antagonist of SR-B1 that increases HDL levels, competitively inhibited infection of HEX-293T by the virus (Wei et al., 2020), thus providing another avenue of possible prophylactic intervention. Interestingly, these authors analyzed the sequences of SARS-CoV-proteins and found the presence of 6 cholesterol recognition motifs like the ones we have described in membrane proteins (Fantini and Barrantes, 2013), including the adjacent mirror CARC-CRAC motif (Fantini et al., 2016).

## Author Contributions

The author confirms being the sole contributor of this work and has approved it for publication.

## Conflict of Interest

The author declares that the research was conducted in the absence of any commercial or financial relationships that could be construed as a potential conflict of interest.
